# Inter-observer agreement of preoperative cardiopulmonary exercise test interpretation in major abdominal surgery

**DOI:** 10.1186/s12871-022-01680-y

**Published:** 2022-04-30

**Authors:** Ruud F. W. Franssen, Anne J. J. Eversdijk, Mayella Kuikhoven, Joost M. Klaase, F. Jeroen Vogelaar, Maryska L. G. Janssen-Heijnen, Bart C. Bongers

**Affiliations:** 1 Department of Clinical Physical Therapy, VieCuri Medical Center, Tegelseweg 210, 5912 BL Venlo, the Netherlands; 2 Department of Epidemiology, GROW School for Oncology and Developmental Biology, Faculty of Health, Medicine and Life Sciences, Maastricht University, Maastricht, the Netherlands; 3 Department of Sports Medicine, VieCuri Medical Center, Venlo, the Netherlands; 4 Fanaticus Sports Medicine, Sports Medical Center, Groningen and Arnhem, the Netherlands; 5 Department of Hepatopancreatobiliary Surgery and Liver Transplantation, University Medical Center Groningen, Groningen, the Netherlands; 6 Department of Surgery, VieCuri Medical Center, Venlo, the Netherlands; 7 Department of Epidemiology, VieCuri Medical Center, Venlo, the Netherlands; 8 Department of Epidemiology, Care and Public Health Research Institute (CAPHRI), Faculty of Health, Medicine and Life Sciences, Maastricht University, Maastricht, the Netherlands; 9 Department of Nutrition and Movement Sciences, School of Nutrition and Translational Research in Metabolism (NUTRIM), Faculty of Health, Medicine and Life Sciences, Maastricht University, Maastricht, the Netherlands

**Keywords:** Exercise testing, Preoperative risk assessment, Prehabilitation, Abdominal surgery, Preoperative evaluation

## Abstract

**Background:**

Accurate determination of cardiopulmonary exercise test (CPET) derived parameters is essential to allow for uniform preoperative risk assessment. The objective of this prospective observational study was to evaluate the inter-observer agreement of preoperative CPET-derived variables by comparing a self-preferred approach with a systematic guideline-based approach.

**Methods:**

Twenty-six professionals from multiple centers across the Netherlands interpreted 12 preoperative CPETs of patients scheduled for hepatopancreatobiliary surgery. Outcome parameters of interest were oxygen uptake at the ventilatory anaerobic threshold (V̇O_2VAT_) and at peak exercise (V̇O_2peak_), the slope of the relationship between the minute ventilation and carbon dioxide production (V̇E/V̇CO_2_-slope), and the oxygen uptake efficiency slope (OUES). Inter-observer agreement of the self-preferred approach and the guideline-based approach was quantified by means of the intra-class correlation coefficient.

**Results:**

Across the complete cohort, inter-observer agreement intraclass correlation coefficient (ICC) was 0.76 (95% confidence interval (CI) 0.57–0.93) for V̇O_2VAT_, 0.98 (95% CI 0.95–0.99) for V̇O_2peak_, and 0.86 (95% CI 0.75–0.95) for the V̇E/V̇CO_2_-slope when using the self-preferred approach. By using a systematic guideline-based approach, ICCs were 0.88 (95% CI 0.74–0.97) for V̇O_2VAT_, 0.99 (95% CI 0.99–1.00) for V̇O_2peak_, 0.97 (95% CI 0.94–0.99) for the V̇E/V̇CO_2_-slope, and 0.98 (95% CI 0.96–0.99) for the OUES.

**Conclusions:**

Inter-observer agreement of numerical values of CPET-derived parameters can be improved by using a systematic guideline-based approach. Effort-independent variables such as the V̇E/V̇CO_2_-slope and the OUES might be useful to further improve uniformity in preoperative risk assessment in addition to, or in case V̇O_2VAT_ and V̇O_2peak_ are not determinable.

**Supplementary Information:**

The online version contains supplementary material available at 10.1186/s12871-022-01680-y.

## Background

There is an increased focus on improving preoperative risk assessment and identification of the high-risk surgical patient scheduled for major surgery in order to guide shared clinical decision-making and patient management [1] by estimating the likelihood of postoperative morbidity and mortality [2]. CPET is an appealing test for preoperative risk assessment, as it provides an objective assessment of the integrative response to exercise of the cardiovascular, pulmonary, and neuromuscular system [3]. Previous research among patients with abdominal cancer has shown that preoperative CPET is an objective and reliable tool for identifying patients at high risk for complications [4–7].

The most frequently reported preoperative CPET-derived parameters that are used for risk assessment in major abdominal surgery are the oxygen uptake (V̇O_2_) at the ventilatory anaerobic threshold (V̇O_2VAT_), the ventilatory equivalent for carbon dioxide (V̇E/V̇CO_2_) at the VAT (V̇E/V̇CO_2VAT_), and the highest attained V̇O_2_ at peak exercise (V̇O_2peak_) [8, 9]. Downsides of these often-used risk assessment parameters are that a maximal effort is required to obtain a valid V̇O_2peak_, which is, depending on the used definition and population, not accomplished in 25–86% of the participants performing CPET [10, 11]. Methods of determining the submaximal V̇O_2VAT_ are complex [12] and there remains controversy about the underlying physiology of the V̇O_2VAT_ [12]. A previous study has shown that the V̇O_2VAT_ is not determinable in approximately 16% of the preoperative CPETs [13].

The use of submaximal indicators of aerobic capacity that are determinable in all patients could improve uniformity and reduce variety of preoperative risk assessment within and between hospitals. The slope describing the relation between minute ventilation and carbon dioxide production (V̇E/V̇CO_2_-slope) is a submaximal parameter of ventilatory efficiency that can be used when V̇E/V̇CO_2VAT_ is not determinable [2]. More recently, the oxygen uptake efficiency slope (OUES) has been introduced as an effort-independent indicator for aerobic capacity in patients undergoing major abdominal surgery [14]. The OUES is well correlated to both V̇O_2VAT_ [14] and V̇O_2peak_ [14, 15].

Although there is some research investigating the inter-observer agreement of the V̇O_2VAT_ and the V̇O_2peak_ in preoperative CPET [13], data on the inter-observer agreement of the preoperative V̇E/V̇CO_2_-slope and OUES are lacking. In addition, it is unknown whether uniformity in determination of CPET-derived parameters can be improved by using a set of guidelines for CPET interpretation. Therefore, the aim of this study was to investigate the inter-observer agreement of determination of preoperative CPET parameters used for preoperative risk assessment in patients undergoing major abdominal surgery by using either a self-preferred or a systematic guideline-based approach.

## Methods

### Study design

In this observational study, **o**bservers representing multiple centers across the Netherlands were asked to interpret 12 preoperative CPETs on two occasions, with at least 4 weeks between each interpretation session. The CPET order was shuffled between the interpretation sessions to prevent observers to be able to recall their previous CPET interpretation. At the first interpretation session, observers interpreted the CPETs using the method(s) they normally use, a self-preferred approach. At the second session, observers used a systematic guideline-based approach for CPET interpretation. The study was approved by the medical ethics committee of Zuyderland (METCZ20200160). Reporting was performed in accordance with the STROBE guidelines for observational studies [16].

### Observers

Potential observers were recruited via the Netherlands Association of Sports Medicine (VSG) and a Dutch network of clinical exercise physiologists and were contacted by e-mail with the request to anonymously fill in a short questionnaire regarding CPET experience, CPET training, preferred CPET interpretation methods, and CPET experience in health-compromised populations. Subsequently, potential observers were asked whether they were potentially willing to participate in a study regarding inter-observer agreement of preoperative CPET interpretation. Potential observers were eligible if they were familiar with interpretation of CPETs in health-compromised populations. All participating observers provided informed consent before taking part in this study.

### Data collection

Preoperative CPETs performed in patients scheduled for hepatopancreatobiliary surgery at the University Medical Centre Groningen were randomly selected from an existing database. The database consisted of CPETs performed on a cycle ergometer (Monark Exercise LC6, Vansbro, Sweden) in upright position using a breath-by-breath CPET system (Quark CPET, COSMED Srl, Rome, Italy) between March 2019 and March 2020. A detailed description of the CPET protocol can be found elsewhere [17]. The CPET protocol comprised a two-minute resting phase, a three-minute warm-up of unloaded cycling, and an incremental phase with constant work rate increments of 5, 10, or 15 W/min, depending on the patient’s estimated physical fitness level and aimed at reaching a maximal effort within eight to 12 min. Throughout CPET, patients had to maintain a pedaling frequency between 60 and 80 revolutions/min. The protocol continued until the patient’s pedaling frequency fell definitely below 60 revolutions/min, despite strong verbal encouragement. Patient data was anonymized and patient characteristics other than date of birth, sex, and body mass were concealed.

All CPETs were interpreted by the observers using the Omnia software version 1.6.8.0 (COSMED Srl, Rome, Italy) that was installed on a remote computer. Data display settings were set to 10-second average fixed time intervals. At least 1 week before each CPET interpretation session, observers received a short software manual. Before each CPET interpretation session, observers were contacted by telephone with oral instructions. In addition, a member of the research team (RF or AE) was available for assistance during each interpretation session. Observers were able to switch between tests as often as desired. During the first interpretation session, observers interpreted the CPETs by using their self-preferred approach. During the second interpretation session, observers used a systematic guideline-based approach for CPET interpretation. The guideline used in this study (see Additional file 1) was composed based on established CPET guidelines [2, 3, 14, 18–20]. Observers were asked to interpret the V̇O_2VAT_, V̇O_2peak_, and V̇E/V̇CO_2_-slope up to the respiratory compensation point on both sessions, whereas they were asked to determine the OUES merely at the second interpretation session as the majority of the observers (73%) appeared not to be familiar with determination of the OUES.

### Statistical analyses

Statistical analyses were performed using IBM SPSS Statistics version 26.0 (IBM, Chicago, IL, USA). A sample size calculation was performed using the sampicc function in STATA statistical software. Based on a previous study of Abbott et al., the estimated intraclass correlation coefficient (ICC) was 0.83 for V̇O_2VAT_ and 0.88 for V̇O_2peak_ [13]. It was hypothesized that the ICC values for the V̇E/V̇CO_2_-slope and OUES would be markedly higher, as interpretation of these parameters is less complex. Starting from an ICC of 0.85 with an estimated full width of the 95% confidence interval (CI) of 0.11 below and above the point estimate, a minimum of 22 raters was required with a sample of 12 CPETs per rater. Descriptive analyses of the data were presented as mean ± standard deviation (SD) or 95% CI, or as median (interquartile range [IQR]), as appropriate based on the Shapiro-Wilk test. Data regarding non-determinable parameters was presented descriptively as percentages relative to the total number of observations per parameter. Inter-observer agreement was estimated for each of the CPETs outcome parameter by calculating the intraclass correlation coefficient (ICC) for the self-preferred approach and the systematic guideline-based approach separately. A two-way random model, single measures and absolute agreement ICC was calculated to estimate the inter-observer agreement. An ICC of 0 indicates no agreement and 1 indicates perfect agreement. ICC values were interpreted according to the classification of reliability, with values < 0.50, 0.50–0.75, 0.75–0.90, and > 0.90 representing poor, moderate, good, and excellent agreement, respectively [21]. In a primary analysis, ICCs of each CPET parameter separately were calculated for the total group of observers. Thereafter, ICCs were calculated for several subgroups of observers.

## Results

A total of 98 completed questionnaires were returned (response rate of 49%), of which 54 responders (55%) agreed to be contacted for further information concerning study participation. Eventually, 27 observers (28%) were willing to participate and provided informed consent. As one observer withdrew before the start of the study, 26 observers (27%) were included in the analyses. There was no loss to follow-up, meaning that all observers completed the 12 CPET observations on both interpretation sessions with a mean ± SD time between interpretation sessions of 66 ± 22 days.

Professions of the participating observers consisted of sports physicians (*n* = 17), sports medicine residents (*n* = 5), and clinical exercise physiologists (*n* = 4). The median [IQR] duration of experience of the observers with CPET interpretation in general and CPET interpretation in health-compromised populations was 7.5 [9.0] and 6.0 [7.0] years, respectively. Observers interpreted 150 [114] CPETs annually (See Table 1).

**Table 1:** Observer characteristics

	n (%)	Median [IQR]]
Sports physician	17 (64.4)	
Sports medicine resident	5 (19.2)	
Clinical exercise physiologist	4 (15.4)	
CPET experience (years)		7.5 [9.0]
Sports physician		10.0 [9.0]
Sports medicine resident		3.0 [2.0]
Clinical exercise physiologist		7.0 [11.0]
CPET experience in health-compromised populations (years)		6.0 [7.0]
Sports physician		7.0 [6.0]
Sports medicine resident		3.0 [2.0]
Clinical exercise physiologist		7.0 [11]
Quantity of observed CPETs annually		150 [114]
Sports physician		150 [100]
Sports medicine resident		100 [247]
Clinical exercise physiologist		226 [277]
Attended a formal CPET course	25 (96)	

*Abbreviations*: *CPET* Cardiopulmonary exercise testing, *IQR* Interquartile range

**Fig. 1:**
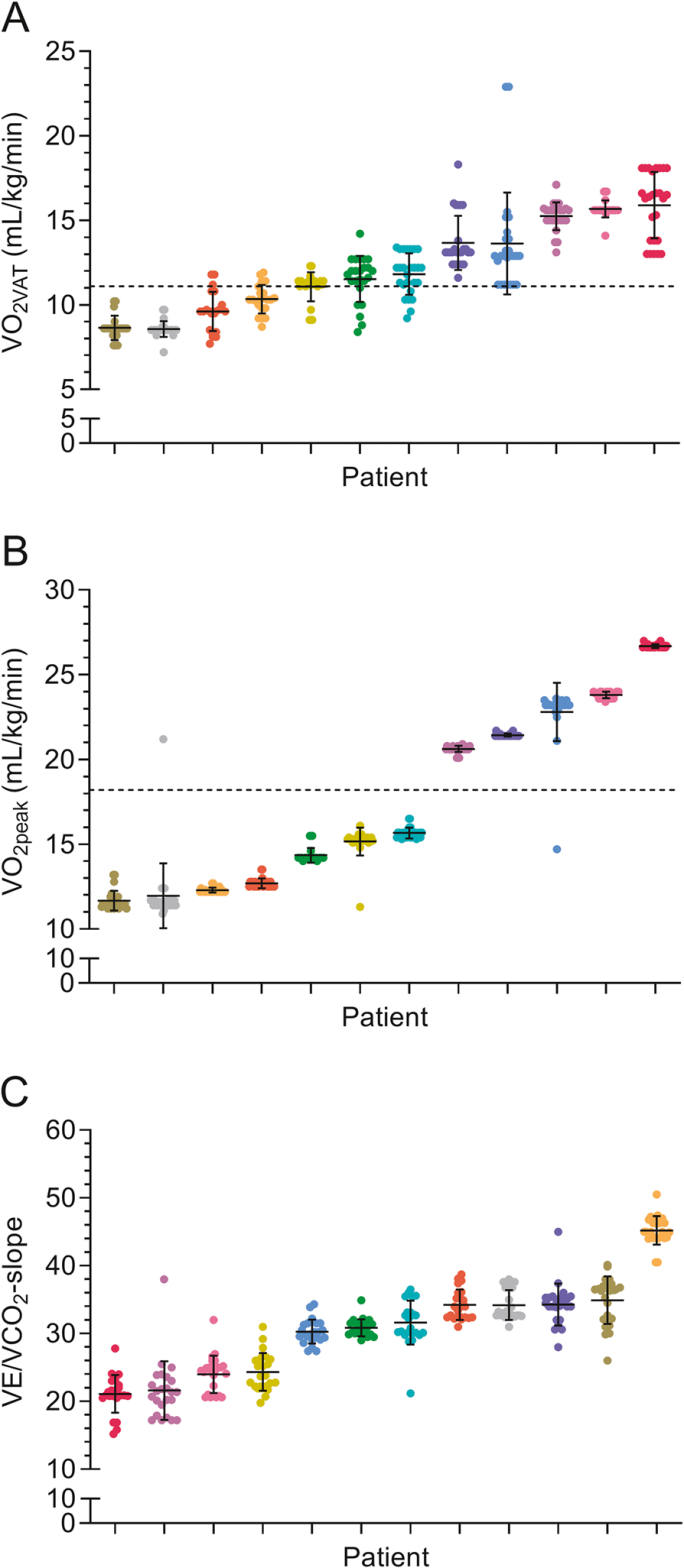
Observed values of the V̇O_2VAT_ (graph A), V̇O_2peak_ (graph B), and V̇E/V̇CO_2_-slope (graph C) in each patient using the self-preferred approach ordered according to increasing value of the mean. Dots represent values determined by individual observers. Each vertical collection of dots represents an individual patient, in which each patient has a unique color throughout all graphs. Horizontal dotted lines represent known risk assessment thresholds defined as 11.1 mL/kg/min for V̇O_2VAT_ [4] (graph A) and 18.2 mL/kg/min for V̇O_2peak_ [4] (graph B). Error bars represent the SD of the mean. Abbreviations: SD = standard deviation; V̇E/V̇CO_2_-slope = slope of the relationship between the minute ventilation and carbon dioxide production; V̇O_2peak_ = oxygen uptake at peak exercise; V̇O_2VAT_ = oxygen uptake at the ventilatory anaerobic threshold

**Fig. 2:**
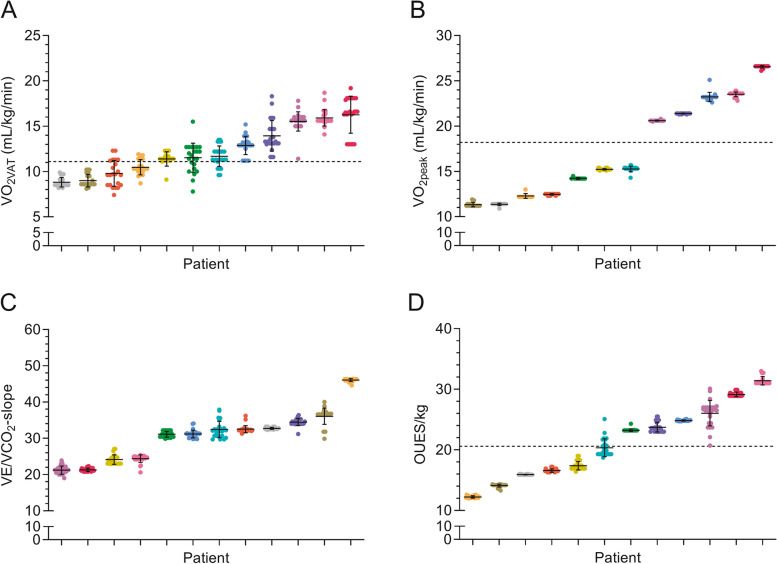
Observed values of the V̇O_2VAT_ (graph A), V̇o_2peak_ (graph B), V̇E/V̇CO_2_-slope (graph C), and OUES/kg (graph D) in each patient using the guideline-based approach ordered according to increasing value of the mean. Dots represent values determined by individual observers. Each vertical collection of dots represents an individual patient, in which each patient has a unique color throughout all graphs. Horizontal dotted lines represent known risk assessment thresholds defined as 11.1 mL/kg/min for V̇O_2VAT_ [4] (graph A), 18.2 mL/kg/min for V̇O_2peak_ [4] (graph B), and 20.6 for the OUES/kg [14] (graph D). Error bars represent the SD of the mean. Abbreviations: OUES = oxygen uptake efficiency slope; SD = standard deviation; V̇E/V̇CO_2_-slope = slope of the relationship between the minute ventilation and carbon dioxide production; V̇O_2peak_ = oxygen uptake at peak exercise; V̇O_2VAT_ = oxygen uptake at the ventilatory anaerobic threshold

The grand mean ± SD of all CPET observations for the complete cohort of observers using the self-preferred and guideline-based approach were respectively 12.1 ± 2.6 and 12.3 ± 2.6 mL/kg/min for V̇O_2VAT_, 17.4 ± 5.3 and 17.3 ± 5.4 mL/kg/min for V̇O_2peak_, and 30.7 ± 6.9 and 30.6 ± 7.1 for the V̇E/V̇CO_2_-slope. The grand mean ± SD OUES normalized for body mass was 21.6 ± 6.1 for all observers using the guideline-based approach. There were no statistically significant differences in determined CPET parameters between the two approaches (See Table 2). Mean values for V̇O_2VAT_, V̇O_2peak_ and the V̇E/V̇CO_2_-slope as interpreted by the observers using both approaches are presented in Table 2 for each interpreted CPET separately. Figure 1 (graph A, B and C) depicts the observed values of the CPET-derived parameters in each patient during the self-preferred approach. Based on the numerical V̇O_2VAT_ and V̇O_2peak_ values reported by the observers, there was no uniform classification whether a patient was considered a low-risk or high-risk patient in respectively 5 and 2 patients (Fig. 1, graph A and B), as observations cross the line identifying the predefined risk thresholds. When using the systematic guideline-based approach, there was no uniform risk classification based on V̇O_2VAT,_ V̇O_2peak_, and the OUES in respectively 5, 0, and 1 patients (see Fig. 2, graph A, B, and D).

**Table 2:** CPET-derived parameters using the self-preferred and guideline-based approach in individual patients

Patient	SPA V̇O_2VAT_ (mL/kg/min)	GBA V̇O_2VAT_ (mL/kg/min)	Number of observations V̇O_2VAT_ (SPA; GBA)	SPA V̇O_2peak_ (mL/kg/min)	GBA Valid V̇O_2peak_^a^ (mL/kg/min)	Number of observations V̇O_2peak_ (SPA; GBA)	SPA V̇E/V̇CO_2_-slope	GBA V̇E/V̇CO_2_-slope	Number of observations V̇E/V̇CO_2_-slope (SPA ^b^; GBA)	GBA OUES/kg	Number of observations OUES (GBA)
1	11.1 ± 0.9	11.4 ± 0.8	26;25	15.2 ± 0.8	15.2 ± 0.1	26;26	24.3 ± 2.8	24.1 ± 1.3	24;26	17.4 (0.7)	26
2	13.6 ± 3.0	12.9 ± 1.0	26;26	22.8 ± 1.7	23.2 ± 0.5	26;18	30.3 ± 1.8	31.2 ± 1.1	24;26	24.8 (0.1)	26
3	9.6 ± 1.2	9.8 ± 1.4	22;22	12.7 ± 0.3	12.5 ± 0.1	26;21	34.2 ± 2.2	32.5 ± 1.0	22 ^c^;26	16.6 (0.3)	26
4	15.9 ± 2.0	16.3 ± 2.1	26;25	26.7 ± 0.1	26.5 ± 0.1	26;26	21.1 ± 2.8	21.3 ± 0.5	24;26	29.2 (0.4)	26
5	11.8 ± 1.2	11.7 ± 1.1	26;26	15.7 ± 0.3	15.3 ± 0.3	26;26	31.6 ± 3.2	32.4 ± 2.3	24;26	20.4 (1.5)	26
6	15.2 ± 0.8	15.5 ± 1.1	26;26	20.6 ± 0.2	20.6 ± 0.0	26;24	21.6 ± 4.3	21.2 ± 1.1	24;26	26.0 (2.1)	26
7	8.6 ± 0.7	9.0 ± 0.7	26;26	11.7 ± 0.6	11.3 ± 0.2	26;18	34.9 ± 3.5	36.1 ± 2.2	24;26	14.1 (0.3)	26
8	15.7 ± 0.5	15.9 ± 0.9	25;26	23.8 ± 0.2	23.5 ± 0.3	26;25	24.0 ± 2.7	24.4 ± 1.0	24;26	31.4 (0.7)	26
9	8.6 ± 0.5	8.8 ± 0.5	26;26	12.0 ± 1.9	11.4 ± 0.1	26;12	34.2 ± 2.2	32.7 ± 0.3	24;26	15.9 (0.0)	26
10	13.7 ± 1.6	13.9 ± 1.7	26;26	21.4 ± 0.1	21.4 ± 0.0	26;22	34.3 ± 3.1	34.5 ± 1.0	24;26	23.7 (0.8)	26
11	10.3 ± 0.8	10.5 ± 0.8	22;22	12.3 ± 0.1	12.3 ± 0.3	26;8	45.2 ± 2.1	46.1 ± 0.4	24;26	12.2 (0.2)	26
12	11.5 ± 1.4	11.5 ± 1.6	24;24	14.4 ± 0.4	14.3 ± 0.1	26;8	30.9 ± 1.3	31.1 ± 0.8	22 ^c^;26	23.3 (0.2)	26
Grand mean	12.1 ± 2.6	12.3 ± 2.6	25;25	17.4 ± 5.3	17.3 ± 5.4	26;20	30.7 ± 6.9	30.6 ± 7.1	24;26	21.6 (6.1)	26
Grand mean difference ^d^ (*P*-value)	−0.2 (*P* = 0.903)			0.2 (*P* = 0.946)			0.1 (*P* = 0.977)				

Values are reported as mean ± SD

^a^Validity of the attained V̇O_2peak_ based on objective criteria of a maximal effort was only determined using the guideline-based approach

^b^Maximum number of observations was 24, as two observers were unfamiliar with interpretation of the V̇E/V̇CO_2_-slope and therefore did not report this parameter

^c^Missing values of unknown origin

^d^Grand mean difference was calculated as SPA minus GBA

*Abbreviations*: *CPET* Cardiopulmonary exercise testing, *GBA* Guideline-based approach, *OUES* Oxygen uptake efficiency slope, *SD* Standard deviation, *SPA* Self-preferred approach, *V̇E/V̇CO*_*2*_*-slope* Slope of the relationship between the minute ventilation and carbon dioxide production, *V̇O*_*2peak*_ Oxygen uptake at peak exercise, *V̇O*_*2VAT*_ Oxygen uptake at the ventilatory anaerobic threshold

### Inter-observer agreement of preoperative CPET interpretation using a self-preferred approach

**Fig. 3:**
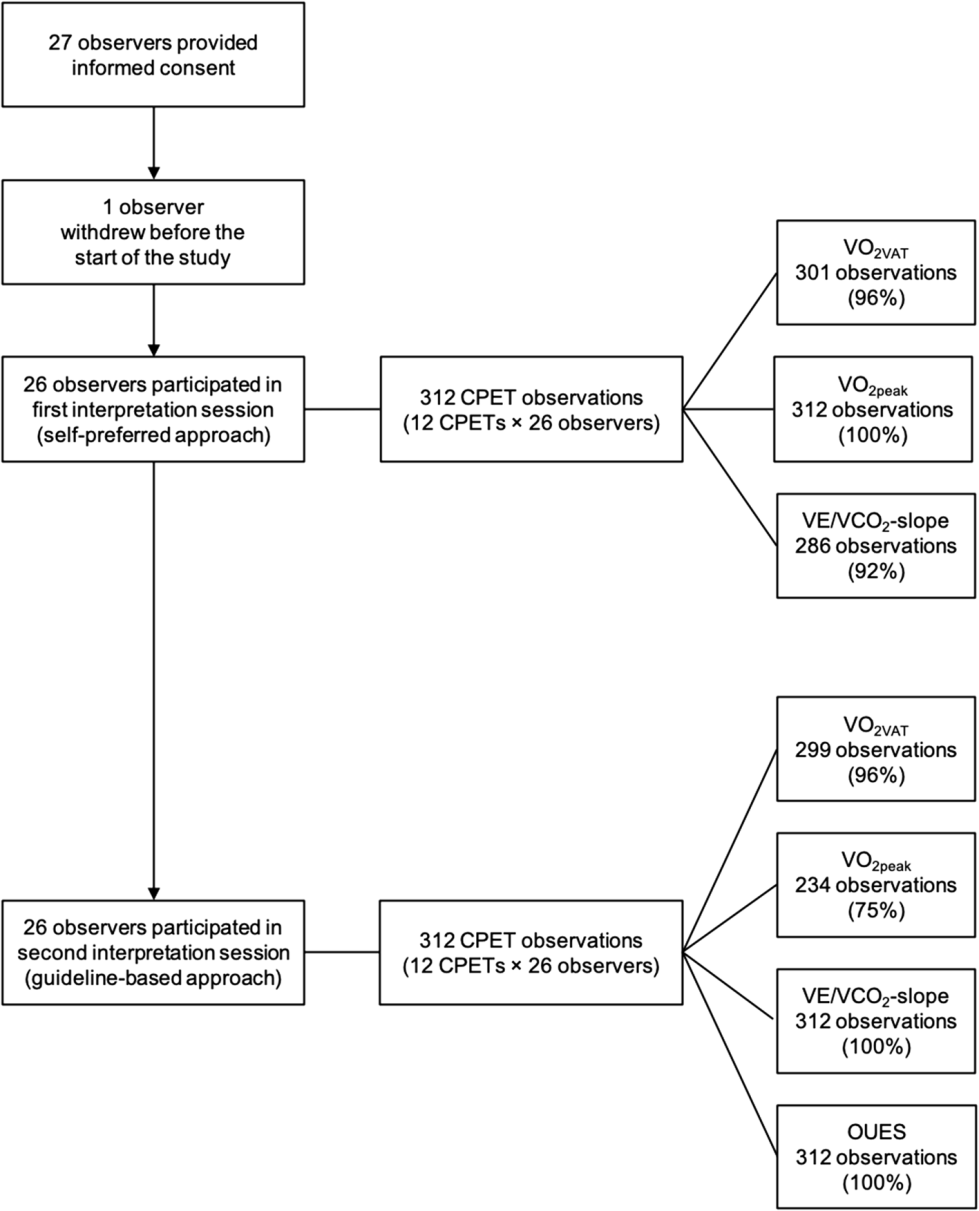
Flow diagram showing the number of study participants (observers) and the total number of observations per CPET-derived parameter for the self-preferred and the systematic guideline-based approach

**Fig. 4:**
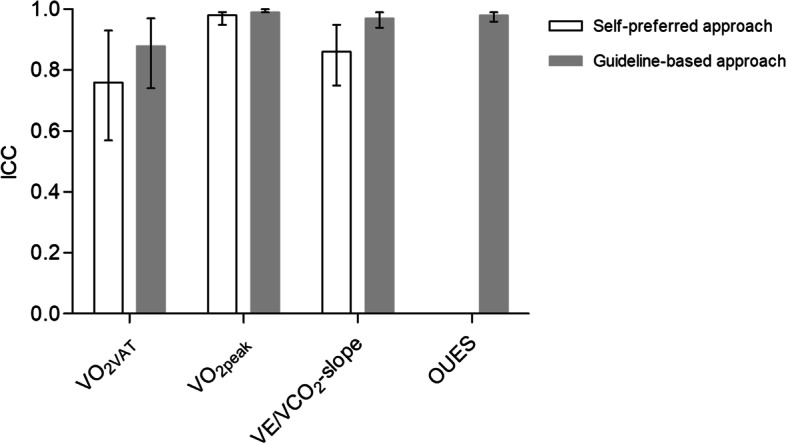
Intra-class correlation coefficient per CPET-derived parameter for the total group of observers. Error bars represent the 95% CI. Abbreviations: CI = confidence interval; OUES = oxygen uptake efficiency slope; V̇E/V̇CO_2_-slope = slope of the relation between the minute ventilation and carbon dioxide production; V̇O_2peak_ = oxygen uptake at peak exercise, V̇O_2VAT_ = oxygen uptake at the ventilatory anaerobic threshold

When using a self-preferred approach, the maximum number of observations per observed CPET parameter was 312 (26 observers × 12 CPETs). Regarding V̇O_2VAT_, 11 (4%) observations were missing, as observers reported them as not determinable. For the V̇E/V̇CO_2_-slope, 26 observations (8.3%) were missing, as two observers (7.8%) were unfamiliar with V̇E/V̇CO_2_-slope interpretation and therefore did not interpret this parameter. In addition, 2 V̇E/V̇CO_2_-slope observations (< 1%) were missing without a known reason. No observations were missing for V̇O_2peak_. See Fig. 3 for an overview of the number of observations per parameter. As depicted in Fig. 4, for the complete cohort of observers, the inter-observer agreement ICC was 0.76 (95% CI 0.57–0.93) for V̇O_2VAT_, 0.98 (95% CI 0.95–0.99) for V̇O_2peak_, and 0.86 (95% 0.75–0.95) for the V̇E/V̇CO_2_-slope. Table 3 shows the inter-observer agreement ICC according to profession, the number of observed CPETs annually, the number of years of experience with CPET interpretation, and the number of years of experience with CPET interpretation in health-compromised populations.

**Table 3:** Inter-observer agreement of CPET-derived parameters in subgroups of observers using the self-preferred and guideline-based approach

	SPA V̇O_2VAT_ ICC (95% CI)	GBA V̇O_2VAT_ ICC (95% CI)	SPA V̇O_2peak_ ICC (95% CI)	GBA V̇O_2peak_^a^ ICC (95% CI)	SPA V̇E/V̇CO_2_-slope ICC (95% CI)	GBA V̇E/V̇CO_2_-slope ICC (95% CI)	GBA OUES ^b^ ICC (95% CI)
Profession							
Sports physician (*n* = 17)	0.77 (0.57–0.93)	0.87 (0.74–0.97)	0.97 (0.99–1.0)	1.00 (1.00–1.00)	0.83 (0.70–0.94)	0.97 (0.94–0.99)	0.99 (0.97–0.99)
Sports medicine residents (*n* = 5)	0.83 (0.66–0.94)	0.87 (0.71–0.97)	0.99 (0.99–1.00)	1.00 (0.99–1.00)	0.87 (0.75–0.96)	0.97 (0.97–1.00)	0.98 (0.96–0.99)
Clinical exercise physiologist (*n* = 4)	0.66 (0.35–0.89)	0.76 (0.51–0.91)	0.87 (0.73–0.96)	0.99 (0.97–1.00)	0.97 (0.93–0.99)	0.97 (0.93–0.99)	0.97 (0.92–0.99)
CPET experience							
≤ 7 years (*n* = 13)	0.81 (0.63–0.94)	0.87 (0.72–0.97)	0.98 (0.95–0.99)	1.00 (0.99–1.00)	0.88 (0.77–0.96)	0.98 (0.95–0.99)	0.99 (0.97–0.99)
7 years (*n* = 13)	0.72 (0.50–0.92)	0.86 (0.71–0.96)	0.98 (0.95–0.99)	1.00 (1.00–1.00)	0.84 (0.71–0.94)	0.97 (0.93–0.99)	0.98 (0.95–0.99)
CPET experience in health-compromised populations							
≤ 6 years (*n* = 12)	0.78 (0.60–0.92)	0.88 (0.75–0.97)	0.98 (0.95–0.99)	1.00 (1.00–1.00)	0.90 (0.82–0.97)	0.98 (0.96–0.99)	0.98 (0.97–1.00)
> 6 years (*n* = 14)	0.75 (0.54–0.93)	0.83 (0.67–0.95)	0.98 (0.95–0.99)	1.00 (1.00–1.00)	0.82 (0.67–0.93)	0.96 (0.92–0.99)	0.97 (0.96–0.99)
Number of CPETs interpreted annually							
≤ 150 (*n* = 14)	0.75 (0.54–0.93)	0.88 (0.74–0.97)	1.00 (0.99–1.00)	1.00 (1.00–1.00)	0.82 (0.67–0.93)	0.98 (0.96–0.99)	0.98 (0.97–0.99)
> 150 (*n* = 12)	0.79 (0.62–0.93)	0.83 (0.69–0.94)	0.95 (0.90–0.98)	1.00 (0.99–1.00)	0.90 (0.81–0.97)	0.96 (0.92–0.99)	0.98 (0.95–0.99)

^a^Interpret with caution, as ICC values are based on a small number of valid observations

^b^Only determined by using the guideline-based approach

*Abbreviations*: *CI* Confidence interval, *CPET* Cardiopulmonary exercise testing, *GBA* Guideline-based approach, *ICC* Intraclass correlation coefficient, *OUES* Oxygen uptake efficiency slope, *SPA* Self-preferred approach, *V̇E/V̇CO*_*2*_*-slope* Slope of the relationship between the minute ventilation and carbon dioxide production, *V̇O*_*2peak*_ Oxygen uptake at peak exercise, *V̇O*_*2VAT*_ Oxygen uptake at the ventilatory anaerobic threshold

### Inter-observer agreement of preoperative CPET interpretation using a guideline-based approach

As there was no loss to follow-up of observers, the maximum number of observations when using a guideline-based approach also was 312 observations per CPET parameter. For V̇O_2VAT_, 13 observations (4%) were missing due to observers reporting the parameter as undeterminable. For V̇O_2peak_, 78 observations (25%) were missing because observers reported that no valid V̇O_2peak_ could be determined. Regarding the V̇E/V̇CO_2_-slope and OUES, no observations were missing. Figure 3 depicts an overview of the number of observations per parameter. As depicted in Fig. 4, for the complete cohort of observers, the inter-observer agreement ICC for V̇O_2VAT_ was 0.88 (95% CI 0.74–0.97), 0.99 (95% CI 0.99–1.00) for V̇O_2peak_, 0.97 (95% CI 0.94–0.99) for the V̇E/V̇CO_2_-slope, and 0.98 (95% CI 0.96–0.99) for the OUES. Table 3 shows the inter-observer agreement ICC categorized according to profession, the number of observed CPETs annually, the number of years of experience with CPET interpretation, and the number of years of experience with CPET interpretation in health-compromised populations. There were no significant differences between categories.

## Discussion

The aim of the current study was to determine the inter-observer agreement of preoperative CPET-derived risk assessment parameters by using either a self-preferred approach or a systematic guideline-based approach. When using a self-preferred approach, inter-observer agreement within the whole cohort of observers was moderate-to-good for V̇O_2VAT_, excellent for V̇O_2peak_, and good for the V̇E/V̇CO_2_-slope. Inter-observer agreement when using a guideline-based approach was good for V̇O_2VAT_ and excellent for V̇O_2peak_, the V̇E/V̇CO_2_-slope, and the OUES. This implies that inter-observer agreement of CPET-derived parameters might be improved by using a systematic guideline-based approach. These findings are important for improvement of preoperative risk assessment and future clinical guideline development.

High levels of inter-observer agreement are paramount to allow for reliable and uniform preoperative risk assessment to guide shared clinical decision-making and optimize patient management. V̇O_2VAT_ and V̇O_2peak_ are generally considered to be the most important preoperative risk assessment parameters that are consistently and independently associated with postoperative outcomes following major abdominal surgery [8]. The ICC value for the determined V̇O_2VAT_ using the self-preferred approach found in the current study was lower than the previously reported inter-observer agreement ICC value for V̇O _VAT_ in the United Kingdom (0.76 versus 0.83 respectively) [13]. On the contrary, the ICC value for V̇O_2peak_ was higher in the current study compared to the UK study (0.98 versus 0.88, respectively). The lower ICCs for V̇O_2VAT_ found in the current study might be a reflection of the less extensive utilization of preoperative CPET and less uniformity of preoperative CPET interpretation and training in the Netherlands compared to the UK. The latter probably affects the inter-observer agreement of V̇O_2VAT_ to a greater extent than V̇O_2peak_**,** as methods for determining V̇O_2VAT_ are more complex than methods for V̇O_2peak_ determination [12].

Besides variation coming from inter-observer (dis) agreement, also other sources that add variability to the reported numerical values of CPET-derived parameters should be considered to improve uniformity of preoperative risk assessment. Other than inter-observer variation, data display methods, the used CPET protocol, measurement error, and within-patient physiological variation, are examples of sources that add variability to CPET-derived parameters. Although the present study showed that inter-observer agreement of V̇O_2VAT_ is good when using a systematic guideline-based approach, variation coming from other sources also needs to be minimized to allow for adequate and reliable preoperative risk assessment. In addition, taking these different sources of variation into account, a V̇O_2VAT_ of 10.9 mL/kg/min (considered a high-risk patient) in reality is probably not much different from an V̇O_2VAT_ of 11.3 mL/kg/min (considered a low-risk patient) [22]. As such, even with a good inter-observer agreement, perhaps less rigid thresholds should be considered for risk assessment as was already proposed by Rose et al. [23].

To improve inter-observer agreement and to allow for adequate and a more uniform preoperative risk assessment, more solid parameters that are identifiable in all patients, such as the V̇E/V̇CO_2_-slope and the OUES might be of added value. The V̇E/V̇CO_2_-slope is an effort-independent parameter that can be used in absence of the more frequently reported preoperative risk assessment parameter V̇E/V̇CO_2VAT_ [24]. The OUES has been reported to be a valid (sub) maximal measure of aerobic capacity in patients undergoing colorectal surgery, and its predictive ability indicates that it might help discriminate patients at higher risk for postoperative complications [14]. Additionally, the OUES has been found to have excellent test-retest reliability in general surgical patients [25]. The ICC of the V̇E/V̇CO_2_-slope and the OUES in our study was excellent and both parameters were objectively determinable in all patients.

The use of the effort-independent variable OUES in preoperative CPET might complement risk assessment, particularly when a parameter (e.g., V̇O_2VAT_) is not determinable, when risk assessment is inconclusive, or when a patient is unable and/or unwilling to deliver a maximal effort. Nevertheless, although the OUES has been directly associated with postoperative complications [26] and mortality [15] in lung cancer patients, there is no evidence concerning a direct association of the preoperative OUES with postoperative complications and mortality in abdominal surgery. More research is needed to elucidate the exact association between the OUES and postoperative outcomes.

The current study has some limitations. First, participating observers were not selected randomly. It is possible that observers who are more confident of their CPET interpretation abilities were more willing to participate in the current study. Although it is difficult to estimate the actual effect of this possible selection bias, this could imply that the inter-observer agreement as presented in the current study might be an overestimation of inter-observer agreement in the total population of observers. Second, some observers (38%) were not familiar with the use of the software. Bias due to observers being not familiar with the software was expected to be minimal as the interpretation software that was used is very user-friendly and easy to comprehend. In addition, we accounted for this by providing a manual and an oral introduction before the start of the CPET interpretation sessions. Moreover, observers were free to switch between tests as much as desired, and a member of the study team was available online at all times to provide immediate assistance when needed. Nevertheless, any software-related bias would probably impact both approaches equally.

Strengths of this study consist of a relatively large number of observers that were willing to participate in both interpretation sessions. There was no loss to follow-up between the two interpretation sessions, meaning that all observers who interpreted the CPETs using the self-preferred approach also interpreted the CPETs using the systematic guideline-based approach. Therefore, differences between the two methods were not reliant on differences in participating observers between sessions.

Future research could focus on the influence of other sources of variation, such as data display intervals on the determination of CPET parameters in order to allow for uniform preoperative risk-assessment. In addition, more research is needed to elucidate the role of the OUES regarding preoperative risk assessment and its direct association with postoperative outcome measures.

## Conclusions

The inter-observer agreement of V̇O_2peak_ is excellent, regardless of the approach that is used. A systematic guideline-based approach can further improve the inter-observer agreement of the numerical values of CPET-derived parameters used for risk assessment. In patients who are unable to achieve a valid V̇O_2peak_, or when V̇O_2VAT_ is not determinable, the V̇E/V̇CO_2_-slope and the OUES could be of added value as these are effort-independent parameters with excellent inter-observer agreement that are determinable in all patients. More research is needed to elucidate the exact role of the V̇E/V̇CO_2_-slope and the OUES within preoperative risk assessment.

## Supplementary Information

**Additional file 1.** Interpretation guidelines. Guideline for systematic interpretation of preoperative cardiopulmonary exercise testing.

## Data Availability

The datasets used and/or analyzed during the current study are available from the corresponding author on reasonable request.

## Abbreviations

CI: Confidence interval
CPET: Cardiopulmonary exercise test
ICC: Intraclass correlation coefficient
IQR: Interquartile range
OUES: Oxygen uptake efficiency slope
V̇E/V̇CO_2_-slope: The slope of the relationship between the minute ventilation and carbon dioxide production
V̇O_2peak_: Oxygen uptake at peak exercise
V̇O_2VAT_: Oxygen uptake at the ventilatory anaerobic threshold
SD: Standard deviation
